# Integration of Liver Glycogen and Triglyceride NMR Isotopomer Analyses Provides a Comprehensive Coverage of Hepatic Glucose and Fructose Metabolism

**DOI:** 10.3390/metabo12111142

**Published:** 2022-11-19

**Authors:** Ivan Viegas, Giada Di Nunzio, Getachew D. Belew, Alejandra N. Torres, João G. Silva, Luis Perpétuo, Cristina Barosa, Ludgero C. Tavares, John G. Jones

**Affiliations:** 1 Centre for Functional Ecology, Department of Life Sciences, University of Coimbra, Coimbra, Portugal; 2 Center for Neurosciences and Cell Biology, University of Coimbra, UC-Biotech, Biocant Park, Nucleo 8, Lote 4, 3060-197 Cantanhede, Portugal; 3 Biotechnology Department, Addis Ababa Science and Technology University, Addis Ababa, Ethiopia; 4 iBiMED, Department of Medical Sciences, University of Aveiro, Aveiro, Portugal; 5 CIVG—Vasco da Gama Research Center, University School Vasco da Gama—EUVG, 3020-210 Coimbra, Portugal

**Keywords:** pentose phosphate pathway, triose phosphates, acetyl-CoA, lipogenesis, ^13^C NMR

## Abstract

Dietary glucose and fructose are both efficiently assimilated by the liver but a comprehensive measurement of this process starting from their conversion to sugar phosphates, involvement of the pentose phosphate pathway (PPP), and conversion to glycogen and lipid storage products, remains incomplete. Mice were fed a chow diet supplemented with 35 g/100 mL drinking water of a 55/45 fructose/glucose mixture for 18 weeks. On the final night, the sugar mixture was enriched with either [U-^13^C]glucose or [U-^13^C]fructose, and deuterated water (^2^H_2_O) was also administered. ^13^C-isotopomers representing newly synthesized hepatic glucose-6-phosphate (glucose-6-P), glycerol-3-phosphate, and lipogenic acetyl-CoA were quantified by ^2^H and ^13^C NMR analysis of post-mortem liver glycogen and triglyceride. These data were applied to a metabolic model covering glucose-6-P, PPP, triose-P, and de novo lipogenesis (DNL) fluxes. The glucose supplement was converted to glucose-6-P via the direct pathway, while the fructose supplement was metabolized by the liver to gluconeogenic triose-P via fructokinase–aldolase–triokinase. Glucose-6-P from all carbohydrate sources accounted for 40–60% of lipogenic acetyl-CoA and 10–12% was oxidized by the pentose phosphate pathway (PPP). The yield of NADPH from PPP flux accounted for a minority (~30%) of the total DNL requirement. In conclusion, this approach integrates measurements of glucose-6-P, PPP, and DNL fluxes to provide a holistic and informative assessment of hepatic glucose and fructose metabolism.

## 1. Introduction

### 1.1. Background

**Figure 1:**
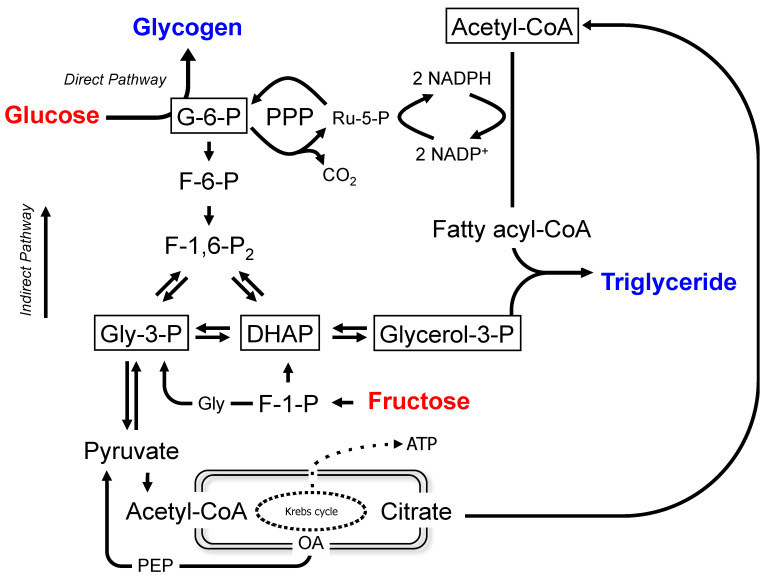
Metabolic model for the synthesis of glycogen and triglyceride from glucose or fructose in the liver. The model includes glucose-6-phosphate oxidation by the pentose phosphate pathway (PPP) to provide NADPH for conversion of acetyl-CoA to fatty acyl-CoA via de novo lipogenesis. The ^13^C-enriched glucose and fructose precursors are highlighted in red and the sampled metabolites, glycogen and triglyceride, are highlighted in blue. The metabolite pools whose ^13^C and ^2^H enrichments are reported by the sampled metabolites, namely, glucose-6-P, triose-P (dihydroxyacetone phosphate and glyceraldehyde 3-phosphate) and lipogenic acetyl-CoA, are highlighted in boxes. Glycogen synthesis from glucose via glucose-6-P from gluconeogenic precursors, including pyruvate and triose-P sources, is also indicated (direct and indirect pathways, respectively). For simplicity, some metabolic intermediates, as well as ATP/ADP and NAD/NADH interconversions, are not shown. Abbreviations are as follows: DHAP—dihydroxyacetone phosphate; F-1-P—fructose-1-phosphate; F-6-P—fructose 6-phosphate; F-1,6-P_2_—fructose-1,6-bisphosphate; G-6-P—glucose 6-phosphate; Gly—glyceraldehyde; Gly-3-P—glyceraldehyde 3-phosphate; OA—oxaloacetate; PEP—phophoenolpyruvate; Ru-5-P: ribulose-5-P.

The liver is a key site for the metabolism of dietary sugar, with glucose and fructose being the principal species absorbed into the portal vein blood outside of milk products. In mammals and many other organisms, the fate of dietary sugar is heavily influenced in real time by systemic glucose homeostasis, with the main priorities being maintenance of a threshold level of blood glucose for the central nervous system and erythrocyte function, while also minimizing large excursions of blood glucose levels. At the same time, sugar is sensed as a precious and desirable nutrient to be sequestered as rapidly and efficiently as possible [1]. This balance is achieved via a highly flexible and well-regulated hepatic metabolic network. Not only can it rapidly switch between net hepatic glucose production and uptake, but it can also direct temporary sugar surplus into short-term storage as glycogen or into longer-term storage as lipids. Since sugar in nature is typically composed of approximately equimolar amounts of glucose and fructose, for omnivorous mammals, including humans, the hepatic metabolic network has evolved to efficiently utilize both hexoses. As can be seen in Figure 1, glucose-6-phosphate (glucose-6-P) is a key nexus in hepatic sugar metabolism since it is a common product of glucose and fructose metabolized via direct and indirect pathways, respectively. Glucose-6-P is also at the intersection of glycogen synthesis and the pentose phosphate pathway (PPP).

The conversion of glucose-6-P to lipids requires the generation of NADPH. The PPP couples the oxidation of glucose-6-P to NADPH generation; hence, in principle, a portion of sugar carbons can be sacrificially oxidized such that the remainder can be converted to lipids. In the liver, NADPH can be derived from other sources [2] and, to the extent that these contribute to de novo lipogenesis (DNL) reducing equivalents, then sugar carbons are spared from PPP oxidation. The PPP is also a conduit for converting hexose sugars to pentose phosphate precursors for nucleotide biosynthesis, which is a continual requirement for hepatocyte growth and turnover.

^13^C-Isotopomers of newly synthesized glycogen derived from [U-^13^C]glucose and [U-^13^C]fructose inform direct and indirect pathway fluxes [3], as well as the fraction of glucose-6-P that underwent PPP oxidation [4]. ^13^C-isotopomers of newly synthesized triglyceride fatty acids and glycerol moieties inform the contributions of these sugars to DNL and glyceroneogenesis [5]. The main objective of this study was to integrate these measurements into a comprehensive description of hepatic glucose and fructose metabolism, starting with their initial phosphorylation to sugar phosphate intermediates and culminating with their conversion to triglycerides. Given the role of excessive sugar consumption and elevated DNL activity in the pathogenesis of non-alcoholic fatty liver disease (NAFLD) [6,7,8], such knowledge will improve our understanding of the role of hepatic glucose and fructose metabolic fluxes in promoting this condition.

### 1.2. Metabolic Model

Figure 1 shows the metabolic model for lipogenesis from glucose and fructose. Fructose is assumed to be converted to triose phosphates via the canonical fructokinase–aldolase–triokinase pathway, while glucose is converted to glucose-6-P via glucokinase. Glucose-6-P can also be synthesized from triose phosphates by gluconeogenesis (GNG). Glucose-6-P is disposed of by conversion to glycogen, by PPP oxidation, and by glycolysis. Glycerol-3-P destined for triglyceride synthesis is mostly derived from the glycolytic triose phosphate pool. The pyruvate product of glycolysis is oxidized to acetyl-CoA, which can be recruited for fatty acid synthesis via DNL. One critical aspect in interpreting the formation of glycogen and triglyceride ^13^C-isotopomers from the ^13^C-glucose or fructose precursors is that turnover of the product pools may not be complete over the duration of the experiment, resulting in artefactual dilutions of glycogen and lipid ^13^C-isotopomer enrichments. To determine the fractions of glycogen and triglyceride that were synthesized while the ^13^C-sugar precursors were present, deuterated water (^2^H_2_O) was administered over the same period. The ^2^H enrichment of glycogen and triglycerides relative to body water informs these fractions [3,5,9] and, by sequential ^2^H and ^13^C NMR analysis, this information can be determined without interfering with the quantification of the ^13^C-isotopomer distributions [3,5,10].

**Figure 2:**
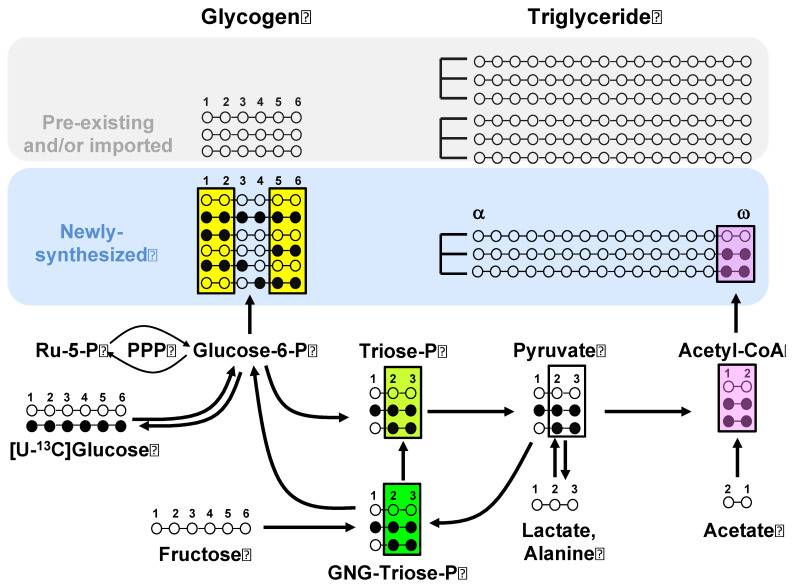
^13^C-Isotopomers of selected metabolic intermediates generated from [U-13C]glucose metabolism into lipogenic and glycogenic pathways. These include hepatic glucose-6-P—inferred from the analysis of newly synthesized glycogen; triose-P recruited for gluconeogenesis (GNG-triose-P)—inferred from the analysis of indirect pathway glycogen 13C-isotopomers; triose-P supplying glycerol-3-P for fatty acid esterification and acetyl-CoA units for de novo lipogenesis—inferred from the 13C-isotopomer analysis of newly synthesized triglyceride glycerol; and the acetyl-CoA pool supplying lipogenesis—inferred from the 13C-isotopomer analysis of newly synthesized fatty acids. For the metabolite carbon skeletons, the filled and unfilled circles represent 13C and 12C, respectively. The shading highlights those 13C-isotopomers that inform the enrichment of the lipogenic acetyl-CoA pool by [U-13C]acetyl CoA from both glycolytic precursor and fatty acid product perspectives, and the colors indicate isotopic enrichment equivalence (same color) or non-equivalence (different colors). For simplicity, in depicting the fatty acid labeling, only the 13C-isotopomers of the last two fatty acid carbons (representing the first acetyl-CoA moiety to be incorporated into de novo lipogenesis) are shown.

Figure 2 shows the principal ^13^C-isotopomers of selected metabolite pools following the metabolism of [U-^13^C]glucose. Under the experimental conditions, the ^13^C-isotopomer distribution of newly synthesized glycogen is assumed to reflect that of glucose-6-P. The direct pathway metabolism of [U-^13^C]glucose generates [U-^13^C]glucose-6-P and the [U-^13^C]glycogen isotopomer. [U-^13^C]Glucose-6-P that undergoes PPP oxidation and recycling generates [1,2-^13^C_2_]glucose-6-P and other partially labeled glucose-6-P isotopomers [4]. In addition, [U-^13^C]glucose that undergoes glycolytic–gluconeogenic recycling (either intrahepatic or via the Cori cycle) generates triose-P isotopomers, principally [1,2,3-^13^C_3_]- and [2,3-^13^C_2_]triose-P [11]. These are incorporated into glucose-6-P and glycogen via GNG, which is also historically referred to as the indirect pathway [12]. The fraction of newly synthesized glycogen derived from the indirect pathway can be estimated from the analysis of its ^2^H enrichment from ^2^H_2_O [3]. Hence, the ^13^C-isotopomer distribution of the GNG precursor pool (GNG-triose-P) can be inferred from that of glycogen after correction for the indirect pathway fraction. Glycerol-3-P for fatty acid esterification is derived from the reduction of dihydroxyacetone phosphate; hence, its ^13^C-isotopomer distribution, read from the analysis of newly synthesized triglyceride glycerol, provides a readout of triose-P ^13^C-isotopomers. Acetyl-CoA isotopomers that are generated from triose-P can be diluted by unlabeled non-triose substrates such as acetate before their incorporation into fatty acids. When the ^13^C-label is provided as [U-^13^C]fructose (Supplementary Figure S1), it generates the same set of hexose and triose-P ^13^C-isotopomers. Note that the formation of [U-^13^C]glucose-6-P from [U-^13^C]fructose can occur via the condensation of [U-^13^C]glyceraldehyde-3-P and [U-^13^C]dihydroxyacetone-P. The probability for [U-^13^C]glucose-6-P formation is related to the fractional enrichments of these triose-P precursors.

## 2. Methods

### 2.1. Materials

[U-^13^C]Fructose at 99% enrichment was obtained from Omicron Biochemicals Inc., South Bend, IN, USA, and [U-^13^C]glucose at 99% enrichment was manufactured by Cambridge Isotopes Limited, Cambridge, MA, USA, and purchased through Tracertec, Madrid, Spain. Deuterated water (^2^H_2_O) at 99.8% was purchased from CortecNet, Les Ulis, France.

### 2.2. Animal Studies

Animal studies were approved by the University of Coimbra Ethics Committee on Animal Studies (ORBEA) and the Portuguese National Authority for Animal Health (DGAV), approval code 0421/000/000/2013. A total of nine adult male C57BL/6J mice obtained from Charles River Labs, Barcelona, Spain, were housed at the University of Coimbra UC-Biotech Bioterium. They were maintained in a well-ventilated environment and a 12 h light/12 h dark cycle. Upon delivery to the Bioterium, mice were provided a two-week interval for acclimation, with free access to water and standard chow, comprising of 60% mixed carbohydrates, 16% protein, and 3% lipids. Following this period, the chow was supplemented with a 55/45 mixture of fructose and glucose present at a concentration of 30% *w*/*v* in the drinking water for a period of 12 weeks. At the beginning of the final evening, mice were administered with an intraperitoneal loading dose of 99% ^2^H_2_O containing 0.9 mg/mL NaCl (4 mL/100 g body weight), and the drinking water was enriched to 5% with ^2^H_2_O. The fructose/glucose mixture in their drinking water was replaced with mixtures of identical composition, but with 20% enriched [U-^13^C]fructose for five mice and 20% enriched [U-^13^C]glucose for the remaining four mice. At the end of this dark cycle, mice were deeply anesthetized with ketamine/xylazine and sacrificed by cardiac puncture. Arterial blood was immediately centrifuged, and plasma was isolated and stored at −80 °C. Livers were freeze-clamped and stored at −80 °C until further analysis.

### 2.3. Analysis of Glycogen and Triglyceride Isotopic Enrichments by NMR

Liver portions of ~500 mg were powdered under liquid nitrogen and extracted with methyl *tert*-butyl ether, as previously described [5]. Glycogen from the insoluble pellet was extracted, purified, and derivatized to monoacetone glucose (MAG), as previously described [3]. Triglycerides from the organic fraction were separated from other lipids, as previously described [13].

#### 2.3.1. NMR Analysis of Glycogen ^2^H and ^13^C-Enrichments

Proton-decoupled ^2^H-NMR spectra of MAG samples at 50 °C were obtained with a Bruker Avance III HD 500 spectrometer using a ^2^H-selective 5 mm probe incorporating a ^19^F-lock channel. Samples were resuspended in 0.5 mL 90% acetonitrile/10% ^2^H-depleted water, to which 50 μL of hexafluorobenzene were added. ^2^H-NMR spectra were obtained with a 90° pulse, 1.6 s of acquisition time, and a 0.1 s interpulse delay. The number of free-induction decays (f.i.d.) collected ranged from 2000 to 10,000. Positional ^2^H enrichments were determined using the MAG methyl signals as an intramolecular standard [14]. To quantify plasma body water ^2^H enrichments, triplicate 10 μL samples of plasma were analyzed at 25 °C by ^2^H NMR, as previously described [15], but with 50 μL of hexafluorobenzene added to the NMR sample. Proton-decoupled ^13^C NMR spectra at 25 °C were obtained with a Varian VNMRS 600 MHz NMR spectrometer equipped with a 3 mm broadband probe. ^13^C NMR spectra were acquired at 25 °C using a 60° pulse, 30.5 kHz spectral width, and 4.1 s of recycling time (4.0 s of acquisition time and 0.1 s pulse delay). The number of acquisitions ranged from 2000 to 18,000. The summed f.i.d. was processed with 0.2 Hz line-broadening and zero-filled to 512 K before Fourier transform.

#### 2.3.2. NMR Analysis of Triglyceride ^2^H and ^13^C Enrichments

Purified triglycerides were dissolved in ~0.5 mL CHCl_3_. To these, 25 μL of a pyrazine standard enriched to 1% with pyrazine-d_4_ and dissolved in CHCl_3_ (0.07 g pyrazine/g CHCl_3_), and 50 μL C_6_F_6_ were added. ^1^H and ^2^H NMR spectra were acquired with an 11.7 T Bruker Avance III HD system using a dedicated 5 mm ^2^H probe with ^19^F lock and ^1^H-decoupling coil, as previously described. ^1^H spectra at 500.1 MHz were acquired with a 90° pulse, 10 kHz spectral width, 3 s acquisition time, and 5 s pulse delay. Overall, 16 f.i.d. were collected for each spectrum. ^2^H NMR spectra at 76.7 MHz were obtained with a 90° pulse, a 1230 Hz sweep width, an acquisition time of 0.67 s, and interpulse delay of 8 s. For ^13^C isotopomer analysis by ^13^C NMR, dried triglyceride samples were dissolved in 0.2 mL 99.96% enriched CDCl_3_ (Sigma-Aldrich) and acquired using the same parameters as for the MAG samples. For each ^13^C spectrum, 2000–4000 f.i.d. were collected.

^13^C and ^2^H NMR spectra were analyzed with ACD/NMR Processor Academic Edition software (ACD/Labs, Advanced Chemistry Development, Inc.).

### 2.4. Estimation of Substrate Contributions to Lipogenesis from Analysis of Newly Synthesized Glycogen and Triglyceride ^13^C Isotopomers

As indicated in Figure 2, the ^13^C-isotopomer distributions of newly synthesized glycogen informs that of glucose-6-P, while the ^13^C-isotopomer distributions of newly synthesized triglyceride glyceryl and fatty acid moieties inform the precursor enrichments of triose-P and lipogenic acetyl-CoA pools, respectively. For each of these reporter metabolites, all ^13^C-isotopomers that are either metabolized to form lipogenic [U-^13^C]acetyl-CoA (i.e., glucose-6-P and triose-P) or are an immediate product (TG-fatty acid) were defined as ^13^C_IUA_. These ^13^C_IUA_ correspond to the shaded ^13^C-isotopomers of glucose-6-P, triose-P, and fatty acids shown in Figure 2 and provide the basis for quantifying the isotopic dilution of the ^13^C-enriched carbons of glucose and fructose as they are metabolized to lipids.

For the glucose-6-P precursors, [U-^13^C]acetyl-CoA can be derived from glycolytic metabolism of [U-^13^C]glucose-6-P, as well as from glucose-6-P isotopomers originating from recycling and/or PPP metabolism of [U-^13^C]glucose. These include [1,2-^13^C_2_]-, [1,2,3-^13^C_3_]-, [5,6-^13^C_2_]-, and [4,5,6-^13^C_3_]glucose-6-P. Thus, as shown by equation (1), the ^13^C*_IUA_* for glucose-6-P can be estimated as the sum of [U-^13^C]-, [1,2-^13^C_2_]-, [1,2,3-^13^C_3_]-, [5,6-^13^C_2_]-, and [4,5,6-^13^C_3_]glucose isotopomer enrichments of glycogen (Σ*_glycogen isotopomers_*) multiplied by 1/*f*_glycogen_. The fraction of newly synthesized glycogen (*f*_glycogen_) is estimated from the ^2^H enrichment of position 2 relative to that of body water [3], and these data are shown in Supplementary Table S2.

Glucose-6-P ^13^C*_IUA_* = Σ*_glycogen isotopomers_* × 1/*f*_glycogen_ (1)

Glucose-6-P is derived from the phosphorylation of dietary glucose and from GNG. For the [U-^13^C]glucose tracer, enrichment of [U-^13^C]glucose-6-P is assumed to be entirely from the direct pathway metabolism of [U-^13^C]glucose. The direct pathway fraction (*f_direct_)*, which also includes sources of unlabeled glucose present in the diet, -can be estimated from the positional ^2^H enrichment distribution of glycogen [3] (Supplementary Table S2). On this basis, ^13^C*_IUA_* enrichment of the dietary glucose precursor pool can be estimated as follows:

Dietary glucose ^13^C*_IUA_* = [U-^13^C]Glucose-6-P ^13^C*_IUA_* × 1/*f_direct_* (2)

Since the fraction of glucose-6-P synthesized by GNG is represented by the indirect pathway fraction of newly synthesized glycogen (*f_indirect_*), which can be estimated from the glycogen ^2^H enrichment distributions (see Supplementary Table S2), then ^13^C*_IUA_* of the GNG precursor pool can be calculated. For the [U-^13^C]glucose tracer, [U-^13^C]glucose-6-P needs to be excluded from Σ*_glycogen isotopomers_* since it is generated via the direct pathway. The glucose-6-P isotopomers formed via gluconeogenesis that can generate [1,2-^13^C_2_]acetyl-CoA are [1,2-^13^C_2_]-, [1,2,3-^13^C_3_]-, [5,6-^13^C_2_]-, and [4,5,6-^13^C_3_]glucose-6-P (^13^C*_IUA-GNG_*):

GNG ^13^C*_IUA_* = Glucose-6-P ^13^C*_IUA-GNG_* × 1/*f_indirect_* (3a)

For [U-^13^C]fructose, all glycogen isotopomers are included since they are by definition all derived via the indirect pathway:

GNG ^13^C*_IUA_* = Glucose-6-P ^13^C*_IUA_* × 1/*f_indirect_* (3b)

The ^13^C*_IUA_* of triose-P and lipogenic acetyl-CoA are estimated by adjustment with the newly synthesized triglyceride glyceryl fraction (*f*_glyceryl_) and fatty acid fractions (*f*_fatty acid_) estimated from the triglyceride ^2^H enrichment distribution [5] (Supplemental Table S2), as follows:

Triose-P ^13^C*_IUA_* = Triglyceride glyceryl ^13^C*_IUA_* × 1/(*f*_glyceryl_) (4)

Acetyl-CoA ^13^C*_IUA_* = Triglyceride fatty acid ^13^C*_IUA_* × 1/(*f*_fatty acid_) (5)

where the measured glyceryl ^13^C*_IUA_* is the sum of triglyceride glyceryl isotopomers with ^13^C in both positions 2 and 3, and the fatty acid ^13^C*_IUA_* is the sum of fatty acid isotopomers with ^13^C in both ultimate (ω) and penultimate positions. The fraction of lipogenic acetyl-CoA derived from triose-P was estimated from the ratio of acetyl-CoA and triose-P ^13^C*_IUA_* as follows:

Triose-P → Acetyl-CoA = 100 × Acetyl-CoA ^13^C*_IUA_*/Triose-P ^13^C*_IUA_* (6)

The fraction of acetyl-CoA derived from non-triose-P metabolites, such as acetate, was estimated as the difference:

Non-triose-P → Acetyl-CoA = 100 − Triose-P fraction(7)

For the mice provided with [U-^13^C]glucose and unlabeled fructose, the fractional contribution of dietary glucose to triose-P was estimated from the ratio of triose-P to dietary glucose ^13^C*_IUA_*. This fraction was adjusted for total lipogenic acetyl-CoA flux by multiplication with the fraction of Acetyl-CoA derived from triose-P (Equation (6)) and for the loss of glucose-6-P carbon 1 as CO_2_ via the PPP.

Dietary glucose → Triose-P = ([100 × Triose-P ^13^C*_IUA_*/dietary glucose ^13^C*_IUA_*] × Equation(6)) + 1/6 PPP (8)

The contribution of gluconeogenic precursors (GNG precursors) to triose-P was calculated as the difference between total triose-P contribution (Equation (6)) and hepatic glucose contribution (Equation (8)) and also accounted for the loss of carbon via the PPP:

GNG precursors → Triose-P = (Equation (6) − Equation (8)) + 1/6 PPP(9)

For the mice provided with [U-^13^C]fructose and unlabeled glucose, the contribution of triose-P to lipogenic acetyl-CoA was estimated using Equation (6). The contribution of GNG precursors to triose-P was estimated from the ratio of triose-P to gluconeogenic triose-P ^13^C*_IUA_*, with adjustment for total lipogenic acetyl-CoA flux by multiplication by Equation (6) and the loss of glucose-6-P carbon 1 during PPP oxidation.

GNG precursors → Triose-P = 100 × (Triose-P ^13^C*_IUA_*/GNG-Triose-P ^13^C*_IUA_*) × Equation (6) + 1/6 PPP (10)

The dietary glucose contribution to triose-P was calculated as the difference between total triose-P (Equation (6)) and the GNG precursor contribution (Equation (10)) and adjusted for the loss of glucose-6-P carbon 1 during PPP oxidation.

Dietary glucose → Triose-P = Equation (6) − Equation (10) + 1/6 PPP(11)

Finally, the contributions of the 20% [U-^13^C]glucose supplement and other unlabeled glucose sources to dietary glucose and the contribution of the 20% [U-^13^C]fructose supplement and other unlabeled gluconeogenic precursors to GNG were calculated as follows:

[U-^13^C]Glucose → dietary glucose = [100 × dietary glucose ^13^C*_IUA_* /20] × Equation (11) (12)

Other glucose sources → dietary glucose = Equation (11) − Equation (12)(13)

[U-^13^C]Fructose → GNG = [100 × GNG precursors ^13^C*_IUA_*/20] × Equation (10) (14)

Other GNG precursors → GNG = Equation (10) − Equation (14)(15)

### 2.5. Estimation of the Fraction of Glucose-6-P Metabolized by the PPP

The fraction of glucose-6-P oxidized by the PPP was estimated from the ^13^C-isotopomer distributions of glycogen, as previously described [4]. The PPP fraction was normalized to total lipogenic acetyl-CoA flux by multiplication with the product of Equation (6).

### 2.6. Statistical Analyses

All results are presented as means ± standard deviations. All datasets were submitted to a Shapiro–Wilk normality test and homoscedasticity test (F test of equality of variances). If both groups presented a normal distribution, then an unpaired Student’s *t*-test was applied (Welch-corrected if variances were unequal). Otherwise, the Mann–Whitney U-test was employed.

## 3. Results

### 3.1. Enrichment of Hepatic Metabolic Pools from [U-^13^C]Glucose and [U-^13^C]Fructose

The ^13^C-isotopomer distributions in the glucose-6-P and triose-P pools were almost all accounted for by ^13^C*_IUA_* species (Supplementary Table S1). For the mice provided with [U-^13^C]glucose, the glucose-6-P pool had the highest ^13^C*_IUA_* abundance, with the principal isotopomer being [U-^13^C]glucose-6-P. From glucose-6-P to glycerol-3-P and acetyl-CoA, there was a stepwise dilution in ^13^C*_IUA_* consistent with an inflow of unlabeled triose-P and acetyl-CoA carbons, respectively (Table 1). The enrichment of the gluconeogenic triose-P pool via indirect pathway metabolism or Cori cycling was relatively low, with the principal contribution coming from PPP activity, as seen by the dominance of [1,2-^13^C_2_]glucose-6-P over that of [5,6-^13^C_2_]glucose-6-P (Supplementary Table S1) [16]. Following its ingestion and subsequent absorption, the [U-^13^C]glucose supplement was diluted almost four-fold by other unlabeled glucose sources by the time it reached the liver (Table 1).

For mice provided with [U-^13^C]fructose, the highest ^13^C*_IUA_* abundances were found in the GNG precursor and triose phosphate pools with dilution at both glucose-6-P and acetyl-CoA pools (Table 1). This enrichment distribution indicates that, under our experimental conditions, fructose was mostly metabolized to triose-P by the liver, followed by carbon flows into both glycogenic and lipogenic pathways. Had the fructose been fully metabolized to glucose in the intestine prior to reaching the liver [17], this would have resulted in a ^13^C*_IUA_* distribution resembling that observed with [U-^13^C]glucose, i.e., highest for glucose-6-P, then progressive dilution at triose-P and acetyl-CoA pools. Finally, in contrast to [U-^13^C]glucose, the dietary [U-^13^C]fructose supplement underwent relatively minor dilution (~1.3-fold) from competing gluconeogenic precursors at its point of entry into the GNG pool.

### 3.2. Sourcing of Lipogenic Acetyl-CoA Carbons Reported by [U-^13^C]Glucose and [U-^13^C]Fructose and PPP Activity

**Figure 3:**
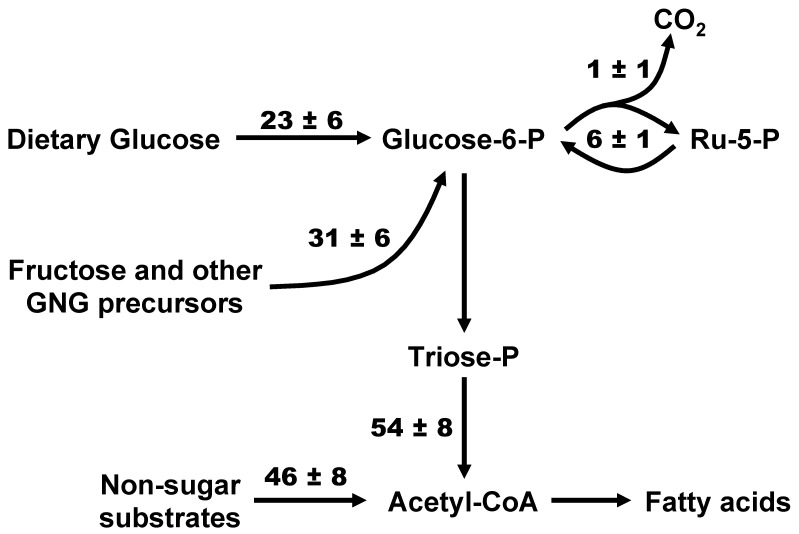
Fractional contributions of sugar and non-sugar sources to lipogenic acetyl-CoA estimated by combining the data of both [U-13C]glucose (n = 4) and [U-13C]fructose (n = 5) analyses. Fractional values were adjusted to that of acetyl-CoA conversion to fatty acids (arbitrarily set to 100) and the standard deviations are shown alongside the means.

A comparison of the contributions of different sources to lipogenic acetyl-CoA estimated from [U-^13^C]glucose and [U-^13^C]fructose tracers is shown in Table 2. Both tracers report a substantial contribution (40–50%) of non-sugar substrates such as acetate to the lipogenic acetyl-CoA pool, even with chronic high-sugar feeding. Under our study conditions, the bulk of triose-P destined for lipogenesis was derived from either dietary glucose or fructose, with only minor contributions from other gluconeogenic precursors. For the four common component fluxes reported by both tracers, the biggest divergence was found for the triose-P and non-triose-P acetyl-CoA sources, while estimates for the contributions of dietary glucose and GNG precursors to the lipogenic triose-P were in better agreement. Figure 3 shows the values of these fluxes obtained by combining and averaging the data derived from the [U-^13^C]glucose and [U-^13^C]fructose measurements. This includes the overall PPP flux, which represents the sum of PPP fluxes attributed to glucose-6-P derived from dietary glucose (i.e., direct pathway) and glucose-6-P derived from GNG sources (indirect pathway) reported by [U-^13^C]glucose and [U-^13^C]fructose, respectively. Our data indicate that about 11% of glucose-6-P had undergone PPP oxidation. While our previous measurement of fractional PPP utilization of glucose-6-P in these livers showed modest but significant differences between [U-^13^C]glucose and [U-^13^C]fructose tracers [4], the significance was lost after the values were normalized to that of lipogenic acetyl-CoA flux (Table 2).

## 4. Discussion

### 4.1. General Overview

We developed a method for quantifying the major fluxes associated with hepatic sugar metabolism that can be easily applied to mice and other small animal models. We demonstrated that this approach can utilize ^13^C-isotopomer information from either [U-^13^C]glucose or [U-^13^C]fructose. In principle, it could also function with other ^13^C-sugar tracers that have been used as probes of hepatic carbohydrate metabolism such as galactose [18,19] or glycerol [16,20,21]. Alongside the ^2^H_2_O tracer, these can be formulated into the animal’s food or drinking water, allowing hepatic metabolic activity to be measured in unperturbed ad libitum feeding conditions. Although dietary glucose is metabolized by most, if not all, tissues, we can nevertheless identify that which is metabolized first-pass by the liver as intact [U-^13^C]glucose. Paradoxically, although fructose metabolism is more strongly associated with the liver compared to glucose, our metabolic analysis does not provide direct information on hepatic [U-^13^C]fructose prior to it being metabolized to sugar phosphates. This means that, unlike the first-pass hepatic metabolism of [U-^13^C]glucose, we cannot be certain that the observed labeling of hepatic glucose-6-P and triose-P from [U-^13^C]fructose was entirely the result of hepatic [U-^13^C]fructose metabolism.

### 4.2. Hepatic Versus Extrahepatic Fructose Metabolism

The liver was long believed to be the principal site for fructose metabolism, but this has been recently challenged with evidence of other tissues, notably the intestine, with the capacity of enterocytes for fructose phosphorylation and incorporation into glycolytic and gluconeogenic fluxes [17]. Moreover, and perhaps not surprisingly, any fructose that is not immediately absorbed can also be avidly metabolized by the intestinal microbiome [22,23], with products such as acetate being subsequently absorbed and recruited as lipogenic substrates by the liver [23]. As proposed by Jang et al., [17], the extent of intestinal versus hepatic fructose metabolism may be related to the total amount of sugar ingested, with low intakes being accommodated entirely by the intestine, and the liver metabolizing any surplus above and beyond the intestinal capacity for fructose disposal. Our mice were kept for 18 weeks on standard chow that was accompanied by drinking water containing 30 g/100 mL of a 55/45 fructose/glucose mixture. There was no other source of drinking water provided. Assuming a daily water intake of ~7 mL water per mouse [24], this would require ingestion of ~10 mL of the mixture, resulting in about 2.5 g of ingested sugar (1.38 g fructose and 1.12 g glucose). Given the average mouse mass of 35 grams, this translates to 39 g of fructose and 32 g of glucose per kg body mass over 24 h, or an average of ~1.6 g kg^−1^ fructose and ~1.3 g kg^−1^ of glucose per hour. If we compare these quantities to the criteria of low and high-dose sugar intake established by Jang et al. based on single gavages of 0.5 g kg^−1^ and 2 g kg^−1^ of a 1:1 fructose/glucose mixture, respectively [17], then our mice had a sugar intake that was well beyond the high dose defined by Jang et al. Under our study conditions, much, if not most, of the fructose would be expected to be metabolized by the liver, which is consistent with our observed hepatic metabolite ^13^C enrichment patterns from [U-^13^C]fructose.

### 4.3. PPP Flux in Relation to De Novo Lipogenesis

The fraction of glucose-6-P that was oxidized by the PPP was estimated to be 11%. The incorporation of n equivalents of acetyl-CoA into the fatty acid polymer requires 2n-2 equivalents of NADPH; hence, the synthesis of palmitate from 8 acetyl-CoA consumes a total of 14 NADPH. Since two NADPH are generated for each glucose-6-P carbon oxidized to CO_2_ via the PPP, a total of 1.17 glucose-6-P equivalents are required to generate the necessary number of NADPH for the synthesis of each palmitate as follows:

4 Glucose-6-P → 8 Acetyl-CoA → 1 Palmitate

1.17 Glucose-6-P → 14 NADPH → 1 Palmitate

Therefore, if glucose-6-P is the sole contributor of lipogenic acetyl-CoA and if the PPP is the sole source of NADPH, then the fraction of glucose-6-P that is utilized by the PPP relative to the total used for lipogenesis (i.e., PPP oxidation plus acetyl-CoA generation) is 1.17/(4 + 1.17) = 23% (this relationship also approximates for C18 fatty acids: 22.9% versus 22.6% for C16). In adipose tissues, glucose-6-P is considered to be the main precursor of acetyl-CoA [25], with the PPP considered to be the principal source of NADPH [26]. An in situ measurement of PPP flux in human adipose tissue via a microdialysis method yielded a PPP fraction of 17–22%, approaching the theoretical value for quantitative glucose-6-P conversion to fatty acids [27]. In the liver, lipogenic acetyl-CoA is derived from sources other than glucose-6-P, notably acetate. Therefore, under these conditions, if the PPP was the sole source of NADPH, then a higher fractional PPP flux per equivalent of glucose-6-P converted to acetyl-CoA would be required. For example, if acetate and glucose-6-P each contribute 50% of acetyl-CoA for palmitate synthesis as follows:

4 Acetate → 4 Acetyl-CoA

2 Glucose-6-P → 4 Acetyl-CoA

1.17 Glucose-6-P → 14 NADPH

then, to provide the theoretical amount of NADPH, the fraction of glucose-6-P that undergoes PPP oxidation would need to increase to 1.17/(2 + 1.17) = 37%. Our data indicate that glucose-6-P accounted at most for about half of lipogenic acetyl-CoA, but only 11% was oxidized by the PPP. This suggests that the PPP accounted, at the most, for only about 11/37, or about 30%, of the total NADPH demand for DNL under these conditions (If NADPH derived from PPP oxidation was also consumed by other processes, such as the reduction of oxidized glutathione, then its fractional contribution to DNL would be even less than 30%). Other possible sources of cytosolic NADPH include cytosolic NADP-malic enzyme 1 and NADP-isocitrate dehydrogenase 1 [2] and folate-mediated serine catabolism [28].

### 4.4. Limitations of the Approach

There are several important limitations of our approach that must be taken into account when interpreting the results. As previously discussed, our mouse model involved a very high intake of sugar that ensured that the fructose component was predominantly metabolized by the liver. If the amount of sugar was reduced, then it is likely that a much higher proportion of the [U-^13^C]fructose would be metabolized by the intestine to form ^13^C-isotopomers of glucose, lactate, and other metabolites [17], and these would be the principal products seen by the liver rather than [U-^13^C]fructose. Nevertheless, aside from the uncertainty in determining the contribution of fructose to the hepatic gluconeogenic triose-P pool, the ^13^C-isotopomer distributions of glycogen and triglycerides would still provide valid information on PPP fluxes, glyceroneogenesis, and the contribution of glucose-6-P and non-glucose-6-P sources to DNL. Under high sugar intake conditions, Jang et al. reported a substantial amount of fructose metabolism by the intestinal microbiota [17], with acetate being a principal product [23]. The microbial fermentation of [U-^13^C]fructose results in the formation of [U-^13^C]acetate, whose incorporation into DNL is indistinguishable from that of [U-^13^C]acetyl-CoA derived from hepatic [U-^13^C]fructose metabolism. To the extent that the fermentative metabolism of [U-^13^C]fructose contributes to the fatty acid ^13^C-isotopomer enrichment, then the fraction of acetyl-CoA derived from non-glucose-6-P sources would be expected to be underestimated, and, accordingly, the contribution of glucose-6-P to DNL overestimated. However, when these parameters obtained from [U-^13^C]fructose are compared with those derived from [U-^13^C]glucose (Table 2), they show a strong tendency to report higher non-glucose-6-P and lower glucose-6-P fractions. One possibility is that, given the very high sugar intake, there may have also been extensive microbial metabolism of [U-^13^C]glucose. Glucose is normally efficiently absorbed in the small intestine, but small intestinal bacterial overgrowth [29,30], possibly induced by high sugar diets [31], can result in a portion of the glucose being fermented instead. Finally, the PPP flux is based on the sugar phosphates that are recycled back to fructose-6-P and glucose-6-P and does not take into account those pentose-P equivalents that were recruited for nucleotide biosynthesis. Thus, the PPP estimate represents a lower limit of the real oxidative glucose-6-P flux.

### 4.5. Conclusions

Hepatic metabolism and assimilation of dietary sugar involves the co-ordination of gluconeogenic, glycogenic, PPP, glycolytic, and lipogenic fluxes. While there are longstanding methodologies for measuring these fluxes individually, until now there has been no approach for quantifying fluxes through the entire ensemble. We demonstrate that, with a combination of ^2^H_2_O and a [U-^13^C]hexose sugar that can be either glucose or fructose, these fluxes can be quantified in mice under natural feeding conditions by analysis of liver glycogen and triglyceride ^13^C-isotopomers. In addition to confirming a previous study that a substantial fraction of lipogenic acetyl-CoA is derived from sources other than glucose-6-P, even during high sugar feeding [5], our analysis also reveals that the PPP was not the main supplier of NADPH for DNL, at least under our study conditions. Such information could be valuable in improving our understanding of hepatic sugar metabolism under different physiological and pathophysiological states.

## Figures and Tables

**Table 1:** Fractional enrichments (%) of ^13^C-isotopomers that generate or are associated with lipogenic [U-^13^C]acetyl-CoA (^13^C*_IUA_*) for selected hepatic metabolite pools for a group of four mice provided with ^2^H_2_O and [U-^13^C]glucose tracers, and a group of five mice provided with ^2^H_2_O and [U-^13^C]fructose. Values are reported as means ± SE. N.D. not determined.

	^13^C*_IUA_* (Equation)				
Experiment	Hepatic Glucose-6-P (Equation (1))	Dietary Glucose (Equation (2))	GNG precursors (Equation (3a,b))	Triose-P (Equation (4))	Acetyl-CoA (Equation (5))
[U-^13^C]Glucose and unlabeled fructose (n = 4)	3.78 ± 0.74	5.47 ± 1.25	1.30 ± 0.15	2.50 ± 0.66	1.53 ± 0.43
Unlabeled glucose and [U-^13^C]fructose (n = 5)	5.06 ± 0.34	N.D.	14.99 ± 1.24	8.99 ± 1.05	4.32 ± 0.50

**Table 2:** Estimates of substrate fluxes contributing to lipogenic acetyl-CoA expressed as a fraction of total lipogenic acetyl-CoA flux into fatty acid synthase from ^2^H enrichment and ^13^C-isotopomer analysis of a group of mice provided with ^2^H_2_O and [U-^13^C]glucose tracers (n = 4), and a group provided with ^2^H_2_O and [U-^13^C]fructose (n = 5). The estimated pentose phosphate pathway (PPP) fluxes involved in glucose-6-P oxidation and carbon recycling to regenerate glucose-6-P (Glucose-6-P → PPP → Glucose-6-P) are also shown.

Pathway Component		[U-^13^C]Glucose	[U-^13^C]Fructose	*p* Value
Acetyl-CoA → Fatty acids		100	100	N.D.
Non-Triose-P → Acetyl-CoA	Equation (7)	40 ± 4	51 ± 8	0.08
Triose-P → Acetyl-CoA	Equation (6)	60 ± 4	49 ± 8	0.08
Dietary glucose → Triose-P	Equations (8) and (11)	28 ± 4	21 ± 9	0.32
[U-^13^C]glucose → Dietary glucose	Equation (12)	8 ± 4	N.D.	N.D.
Other dietary glucose sources → Dietary glucose	Equation (13)	19 ± 4	N.D.	N.D.
GNG precursors → Triose-P	Equations (9) and (10)	34 ± 8	29 ± 4	0.38
[U-^13^C]fructose → GNG	Equation (14)	N.D.	22 ± 6	N.D.
Other precursors → GNG	Equation (15)	N.D.	7 ± 4	N.D.
Glucose-6-P → PPP → Glucose-6-P		7 ± 1	5 ± 1	0.13

Values are reported as means ± SD. N.D. not determined.

## Data Availability

The datasets generated during and/or analyzed during the current study are available from the corresponding author on reasonable request. The data are not publicly available due to privacy.

## Supplementary Materials

The following supporting information can be downloaded at: https://www.mdpi.com/article/10.3390/metabo12111142/s1, Figure S1: ^13^C-Isotopomers of selected metabolic intermediates generated from [U-^13^C]fructose metabolism into lipogenic and glycogenic pathways. These include hepatic glucose-6-P—inferred from the analysis of newly-synthesized glycogen; triose-P recruited for gluconeogenesis (GNG-triose-P)—inferred from the analysis of indirect pathway glycogen ^13^C-isotopomers; triose-P supplying glycerol-3-P for fatty acid esterification and acetyl-CoA units for de novo lipogenesis—inferred from the ^13^C-isotopomer analysis of newly-synthesized triglyceride glycerol, and the acetyl-CoA pool supplying lipogenesis—inferred from the ^13^C-isotopomer analysis of newly-synthesized fatty acids. For the metabolite carbon skeletons, the red filled and unfilled circles represent ^13^C and ^12^C, respectively. The shading highlights those isotopomers that form [U-^13^C]acetyl CoA and the colors indicate isotopic equivalence (same color) or non-equivalence (different colors). For simplicity, in depicting the fatty acid labeling, only the ^13^C-isotopomers of the last two fatty acid carbons (representing the first acetyl-CoA moiety to be incorporated into DNL) are shown.; Table S1: Liver glycogen ^13^C-isotopomer enrichments from mice provided with [U-^13^C]glucose (*n* = 4) and [U-^13^C]fructose (*n* = 5). The glycogen ^13^C-isotopomers shown in bold text are metabolized to [U-^13^C]acetyl-CoA. Table S2: Newly synthesized glycogen fraction (*f*_glycogen_) with direct and indirect pathway contributions to the newly synthesized glycogen (*f_direct_* and *f_indirect_*), and newly synthesized triglyceride glyceryl and fatty acid fractions (*f*_glyceryl_ and *f*_fatty acid_) from ^2^H-enrichment data of liver glycogen and triglyceride, respectively.
