# Supplementary material for: Integration of Liver Glycogen and Triglyceride NMR Isotopomer Analyses Provides a Comprehensive Coverage of Hepatic Glucose and Fructose Metabolism
Source: Metabolites. 2022 Nov 19;12(11):1142. doi: 10.3390/metabo12111142 (PMC9698123; doi:10.3390/metabo12111142)
Supplement: Supplementary file 1 [file metabolites-12-01142-s001.zip › metabolites-2000371-supplementary.pdf]

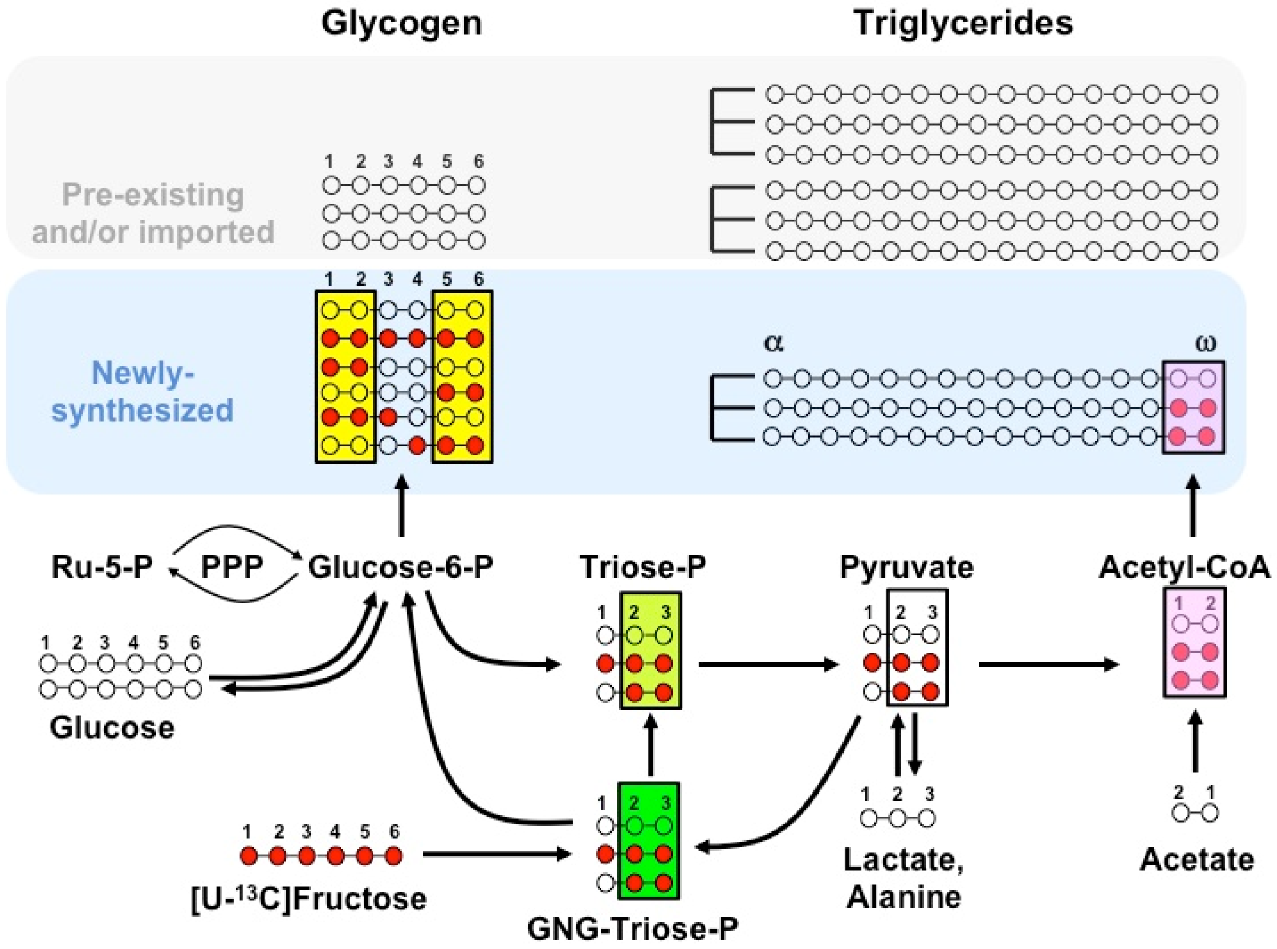

Supplementary Figure S1: ^{13}C-Isotopomers of selected metabolic intermediates generated from [U-^{13}C]fructose metabolism into lipogenic and glycogenic pathways. These include hepatic glucose-6-P - inferred from the analysis of newly-synthesized glycogen; triose-P recruited for gluconeogenesis (GNG-triose-P) – inferred from the analysis of indirect pathway glycogen ^{13}C-isotopomers; triose-P supplying glycerol-3-P for fatty acid esterification and acetyl-CoA units for de novo lipogenesis – inferred from the ^{13}C-isotopomer analysis of newly-synthesized triglyceride glycerol, and the acetyl-CoA pool supplying lipogenesis – inferred from the ^{13}C-isotopomer analysis of newly-synthesized fatty acids. For the metabolite carbon skeletons, the red filled and unfilled circles represent ^{13}C and ^{12}C, respectively. The shading highlights those isotopomers that form [U-^{13}C]acetyl CoA and the colors indicate isotopic equivalence (same color) or non-equivalence (different colors). For simplicity, in depicting the fatty acid labeling, only the ^{13}C-isotopomers of the last two fatty acid carbons (representing the first acetyl-CoA moiety to be incorporated into DNL) are shown.

Supplementary Table S1: Liver glycogen ^{13}C-isotopomer enrichments from mice provided with [U-^{13}C]glucose (*n*=4) and [U-^{13}C]fructose (*n*=5). The glycogen ^{13}C-isotopomers shown in bold text are metabolized to [U-^{13}C]acetyl-CoA. Values are reported as means ± S.D.

**Glycogen ^{13}C-isotopomer**	**[U-^{13}C]Glucose**	**[U-^{13}C]Fructose**
**[U-^{13}C]glycogen**	**2.45 ± 0.84**	**0.56 ± 0.13**
**[1,2,3-$^{13}C_3$]glycogen**	**0.84 ± 0.54**	**1.84 ± 0.49**
**[4,5,6-$^{13}C_3$]glycogen**	**1.03 ± 0.43**	**2.03 ± 0.21**
**[1,2-$^{13}C_2$]glycogen**	**0.38 ± 0.12**	**0.40 ± 0.08**
**[5,6-$^{13}C_2$]glycogen**	**0.03 ± 0.04**	**0.11 ± 0.03**
[2,3-$^{13}C_2$]glycogen	0.02 ± 0.02	0.09 ± 0.02
[4,5-$^{13}C_2$]glycogen	0.02 ± 0.03	0.11 ± 0.03

Supplementary Table S2: Newly synthesized glycogen fraction ($f_{glycogen}$) with direct and indirect pathway contributions to the newly synthesized glycogen (f_{direct} and $f_{indirect}$), and newly synthesized triglyceride glyceryl and fatty acid fractions ($f_{glyceryl}$ and $f_{fatty\ acid}$) from ^{2}H-enrichment data of liver glycogen and triglyceride, respectively. Values are reported as means ± S.D.

^{13}C-tracer	**Glycogen**			**Triglyceride**	
	$f_{glycogen}$	f_{direct}	$f_{indirect}$	$f_{glyceryl}$	$f_{fatty\ acid}$
[U-^{13}C]glucose (n=4)	0.73 ± 0.11	0.62 ± 0.04	0.38 ± 0.04	0.31 ± 0.10	0.16 ± 0.05
[U-^{13}C]fructose (n=5)	0.74 ± 0.08	0.66 ± 0.02	0.34 ± 0.04	0.30 ± 0.07	0.16 ± 0.05
